# Distinct Antibody Fc-profiles in Lymph During Homeostasis and Chronic HIV Infection

**DOI:** 10.20411/pai.v11i1.887

**Published:** 2026-02-23

**Authors:** Ryan P. McNamara, Audrey L. Butler, Sepideh Dolatshahi, Sabian Taylor, Yoav Dori, Ian Frank, Maxim G. Itkin, Michael R. Betts, Galit Alter

**Affiliations:** 1 Ragon Institute of MGH, MIT and Harvard, Cambridge, Massachusetts; 2 Massachusetts Institute of Technology, Cambridge, Massachusetts; 3 Division of Cardiology, Department of Pediatrics, Children’s Hospital of Philadelphia, Philadelphia, Pennsylvania; 4 Division of Infectious Diseases, Department of Medicine, Perelman School of Medicine, University of Pennsylvania, Philadelphia, Pennsylvania; 5 Center for Lymphatic Disorders, Department of Radiology, Perelman School of Medicine, University of Pennsylvania, Philadelphia, Pennsylvania; 6 Department of Microbiology, Perelman School of Medicine, University of Pennsylvania, Philadelphia, Pennsylvania

**Keywords:** Antibodies, Compartmentalization, Fc-receptors, HIV, Isotype, Lymph, Tissues

## Abstract

**Background:**

Antibodies play a critical role in the control of pathogens and tumors through their ability to recognize non-self and then direct immune-mediated destruction. Antibodies are generated by plasma cells or plasmablasts, located throughout the tissues, and are transported between blood, lymph, mucosal secretions, and tissues to survey all sites for pathogens or malignant cells. However, mounting evidence suggests antibodies that transit across compartments (from the blood to the brain, mucosal tissues, or placenta) differ from those in systemic circulation. Whether antibodies also differ as they transit from the blood into non-privileged tissues remains unclear. Thus, here we aimed to define the landscape of antibodies that exist within the blood and tissues and begin to define the properties that lead to antibody transfer across compartments.

**Methods:**

To analyze tissue antibodies, we performed antibody profiling in chyle, a fluid component of lymph collected via the thoracic duct, contrasting these profiles to matched plasma samples.

**Results:**

Equivalent levels of pathogen-specific IgG antibodies and functions were observed across the plasma and lymph in people without HIV. However, this balance in IgG transfer was disrupted in people living with HIV, with significantly lower transfer ratios across several pathogen-specific IgG subpopulations in chyle.

**Conclusion:**

Differential transfer of IgG was Fc-receptor dependent, pointing to a mechanism of transfer into tissues during inflammatory disease that may have a critical role in selecting the antibodies able to access the peripheral and lymphoid tissues.

## INTRODUCTION

While antibodies are primarily implicated in anti-pathogen immunity, they play essential roles in general immune surveillance [1, 2], tumor-clearance [3], commensal-host dynamics [4, 5], as well as in immune regulation [6]. Immune surveillance activities of antibodies rely on their ability to transit throughout circulation and tissues. Antibodies generated in the bone marrow, from tissue-resident plasma cells, or from recently activated plasmablasts, circulate throughout the body via fluid conduits. Thus, immune responses are not isolated to one compartment but require the transport of both immune cells and antibodies [7]. While immune cells can upregulate adhesion molecules in response to inflammatory cues, allowing them to extravasate into tissues, it is thought that antibodies rely on passive processes to enter tissues. B cells may actively gain access to target tissues where they convert to antibody-secreting cells, but antibodies produced distally must transfer into tissues via endothelial barriers.

Antibody transfer across endothelial barriers has been extensively investigated by the vaccine and monoclonal therapeutics fields [8, 9]. While antibody levels and specificities appear to be relatively conserved at mucosal sites compared with the blood [10], significant compartmentalization of antibodies has been documented across the blood-brain barrier [11] and placenta [12]. These data suggest that antibodies rely on highly specialized transport mechanisms or localized production for access to immune-privileged tissues to avoid pathology and inflammation around organs. Emerging data suggest that while the magnitude of the cellular immune response to a pathogen may correlate across blood and tissues, distinct functional profiles exist among tissue-localized immune cells [13–15]. Whether antibodies also possess differential functional capacities upon entering tissues is unknown, but could point to the selective transfer of antibodies and protective functions key to the design of next-generation, targeted monoclonal therapeutics.

In the context of chronic diseases such as HIV or autoimmunity, studies have begun to point to perturbed drug delivery to tissues, arguing that antibody transit into tissues differs during inflammation. For example, in-depth biodistribution analysis of anti-TNF therapeutics has illustrated significantly different abilities of antibodies to penetrate tissues across distinct patient groups [16, 17], arguing that tissues regulate antibody transfer via an active process. Yet, whether antibodies are naturally modified to gain access to tissues/lymphoid sites or whether natural sieving may occur to select for immune-protective, rather than immunopathologic, antibodies remains unclear. To begin to dissect the process by which antibodies may enter into tissues, chyle, a fluid component of lymph collected via the thoracic duct, contains antibodies and can be analyzed to probe antibodies drained from tissues [18]. Chyle is composed of cholesterol, triglycerides, chylomicrons, fat-soluble vitamins, and lymph that drains from all parts of the lower body [19, 20]. Chyle contains immunoglobulins and leukocytes, primarily lymphocytes [15, 20–24], providing a rich source of antibodies from tissues. Yet whether differences exist in the humoral immune profile in chyle and plasma in people living with or without HIV (PLWH or PLWoH, respectively) remains to be defined.

To begin to define whether similar or distinct antibodies exist across the blood and tissues, we profiled antibodies across a wide range of childhood vaccines, endemic pathogens, and HIV in the plasma and chyle of PLWH or PLWoH. The overall magnitude, as well as isotypes, subclasses, Fc receptor (FcR) binding, and Fc effector functions of pathogen antibodies were profiled across both compartments. While similar IgG antibody profiles were observed in the plasma and chyle in PLWoH, IgA and IgM were selectively omitted from the chyle. Moreover, significant differences were observed in IgG transfer into tissues in the setting of HIV infection, marked by changes in FcR-binding profiles in the chyle. These data suggest HIV infection associated inflammation may alter the transfer of antibodies into tissues, which may interfere with both systemic and therapeutic monoclonal antibody-mediated surveillance of infected tissues, and point to a potentially broader defect in antibody tissue access across inflammatory conditions such as HIV, autoimmunity, and cancer.

## METHODS

### Cohort

Matched peripheral blood and TDL samples were collected from PLWoH who were undergoing thoracic duct cannulation for idiopathic or traumatic chylopericardium, chylothorax, and/or chylous ascites (n = 8; University of Pennsylvania or Children’s Hospital of Philadelphia). Additional samples were collected from ART-treated and untreated PLWH with no clinical indication for thoracic duct cannulation via Penn IRB-approved research protocol (n = 12; University of Pennsylvania). Further information on the cohort can be found in Table 1 and described elsewhere [22]. The MGH Institutional Review Boards approved the study, and each participant provided written informed consent for participation in the study.

**Table 1.** Cohort Characteristics

Participant	Age	Sex	Group	Viral Loads (copies/mL)	CD4+T count (mm^3^)
1	27	M	HIV(+) on therapy	52	1315
2	47	M	HIV(+) Not on therapy	281676	381
3	30	M	HIV(+) Not on therapy	73743	416
4	39	M	HIV(+) on therapy	<20	408
5	28	M	HIV(+) on therapy	<20	1391
6	47	M	HIV(+) on therapy	<20	811
7	28	M	HIV(+) on therapy	<20	1062
8	59	M	HIV(+)	Not Reported	Not Reported
9	52	F	HIV(+)	Not Reported	Not Reported
10	35	Not Reported	HIV(+)	Not Reported	Not Reported
11	23	F	HIV(+)	Not Reported	Not Reported
12	65	M	HIV(+)	Not Reported	Not Reported
13	67	M	HIV(−)		
14	Not Reported	Not Reported	HIV(−)		
15	78	F	HIV(−)		
16	11	F	HIV(−)		
17	3	F	HIV(−)		
18	10	F	HIV(−)		
19	61	M	HIV(−)		
20	88	M	HIV(−)		

### Biophysical Profiling

Antigens were acquired from commercial vendors or from collaborators (Supplementary Table 1). Antigen-specific antibody isotyping, subclassing, and FcR binding were measured using a multiplexed bead array technology [25, 26]. Isotype and subclass binding to antigens was done as previously described [27]. PE-conjugated secondary antibodies specific to the antibody isotype (IgM, IgG, IgA) or subclass (IgG1, IgG2, IgG3, IgG4, IgA1, IgA2) were diluted at 1:500 in Luminex Assay Buffer (1X PBS, pH = 7.4, 0.1% BSA, 0.05% Tween-20) to detect the specific antibody feature binding to an antigen. Unbound isotype or subclass-specific antibodies were removed through washing with 1X Assay buffer after 1 hour of incubation at room temperature. Relative levels of the specific isotype and subclasses were calculated as the median fluorescence intensity of PE for that feature.

To prepare FcRs, AVI-Tagged FcRs (variants with the highest prevalence in the population) and FcR were biotinylated with BirA enzyme (Avidity, BirA500) for 30 minutes rotating at room temperature, and excess biotin was removed with Zeba Spin Desalting Columns, 7 MKCO (Thermo Fisher, 89892). Prior to use, biotinylated FcRs were coupled to Streptavidin-PE for 10 minutes. Immune complex binding to FcRs was then measured via flow cytometry.

### Antibody-Dependent Cellular Phagocytosis (ADCP)

Based on the published protocol [28], antigens were biotinylated with LC-LC biotin and coupled to yellow-green fluorescent Neutravidin 1µm beads (Thermo Fisher, F8776) for 2 hours at 37°C. Coupled beads were then washed twice with 5% BSA/PBS and stored at 4°C for up to 1 week. Plasma and chyle samples were diluted 1:100, and a 10 µL sample was incubated with 10 µL antigen-coupled beads for 2 hours at 37°C to form immune complexes. After washing with PBS to remove unbound, non-specific antibody, THP-1 monocytes were added (200 µL/well) at a concentration of 1.25×10^5^ cells/mL and incubated with immune complexes overnight for 16 hours at 37°C. Cells were fixed with 4% PFA and acquired with an Intellicyt iQue. Phagocytosis was measured by a phagocytosis score ((gMFI of bead-positive cells x percentage of bead-positive cells)/1000).

### Antibody-Dependent Neutrophil Phagocytosis (ADNP)

Whole blood was collected from donors through a previously validated approach [29]. Briefly, erythrocytes were lysed from the whole blood using ammonium citrate dextrose (ACD) mixed at a 1:10 ratio with ammonium chloride-potassium (ACK) lysis buffer (150 mM NH_4_Cl, 10 mM KHCO_3_, 0.1 mM Na_2_EDTA, pH = 7.4) at room temperature for 5 minutes. Then, white blood cells were pelleted by centrifugation at 500 * g for 5 minutes at room temperature and washed with 1X phosphate-buffered saline (PBS, pH = 7.4). The neutrophils were then enriched from the white blood cell pellet using the direct human granulocyte isolate kit (StemCell). The cells were seeded into 96-well round-bottomed plates at a concentration of 2.5×10^5^ cells/mL. Separately, pre-immune complexes were formed at 37°C for 2 hours on fluorescent neutravidin microspheres. The microspheres were washed with 1X PBS and then added to the cells to allow for ADNP for 1 hour at 37°C.

After the incubation, cells were stained for CD66b using pacific blue conjugated anti-CD66b for 15 minutes. The mixture was then fixed using 4% paraformaldehyde at room temperature for 15 minutes, and then spun down at 500 * g at 4°C for 5 minutes. The fixed cells were washed twice with ice cold 1X PBS. Cells were then analyzed via flow cytometry using CD66+ to gate for neutrophils. ADNP was quantified via PhagoScore as previously described [29].

### Antibody-Dependent Complement Deposition (ADCD)

Based on the published protocol [30], antigens were biotinylated and coupled to 1 μm red fluorescent neutravidin microspheres (Fluospheres, ThermoFisher) as described above. Pre-immune complexes were allowed to form with the antigen-coated microspheres for 2 hours at 37°C. The samples were diluted at 1:10 in 1X PBS. Lyophilized guinea peig complement was resuspended in water, and was then added to gelatin veronal buffer supplemented with Mg2+ and Ca2+. The complement solution was then added to each well containing the pre-immune complexes and allowed to incubate at 37°C for 50 minutes. After the incubation, plates were spun down at 500 * g for 5 minutes, supernant was removed, and the reaction was quenched through the addition of 200 µL of 15 mM EDTA per well. The plates were then spun down and washed twice with 1X PBS, and excess supernatant was discarded after each spin.

For detection of deposited complement, anti-guinea pig C3-FITC was added to each well. The plates were allowed to incubate at room temperature for 15 minutes for staining. The plates were then washed with 1X PBS to remove unbound detection antibody. Samples were analyzed on the iQue Screener PLUS platform (Intellicyt). Data was analyzed using ForeCyt software (Intellicyt) and recorded as median fluorescent intensity of FITC.

### Statistics and Multivariate Analyses

Statistical comparisons between chyle and blood measurements were computed using a paired nonparametric t-test. Comparisons across HIV-status groups were computed using an unpaired nonparametric t-test. All tests were corrected for multiple comparisons using a Bonferroni’s correction. Correlations between chyle and blood IgG levels and FcR binding were performed in GraphPad Prism as Spearman correlations, *P*<0.0001. Principal Component Analysis was per-formed using JMP software. To identify the key features contributing to the profile differences between chyle and blood, Multi-Level Partial Squares Discriminant Analysis (MLPSDA) [12, 31–33] was performed. These models were constructed using antigen-specific IgG1s and FcRs after z-scoring the measurements for HIV-positive and HIV-negative samples separately. Variables were centered and scaled to a standard deviation of 1. Five-fold cross-validation was performed on the data (Venetian blinds) and Cross Validation (CV) accuracies were reported. To additionally assess model significance, a permutation test was performed by randomly shuffling labels. The MLPLSDA model performance was reported in terms of *P*-values assessing if the model performed better than label-shuffled random models (Wilcoxon *P* values).

## RESULTS

### Coordination of Functional IgG, But Not Other Isotypes, Between Chyle and Blood

Antibody selection occurs across several barriers, including the blood-brain barrier and the placenta [2, 34, 35]. However, whether antibodies survey the blood and non-privileged tissues equally is unclear. Using chyle as a source of lymphatic fluid, which drains the body’s tissues, we initially broadly profiled antigen-specific antibody isotypes (IgG, IgM, IgA), IgG subclasses (1, 2, 3, 4), FcR binding (FcγRIIIA, IIIB, IIA, IIB, FcRn), and antibody-dependent innate effector functions (monocyte and neutrophil phagocytosis, and complement deposition) across a variety of different vaccine and/or endemic viruses and bacteria across 8 PLWoH participants who ranged in age from 3 to 88 years.

**Figure 1.**
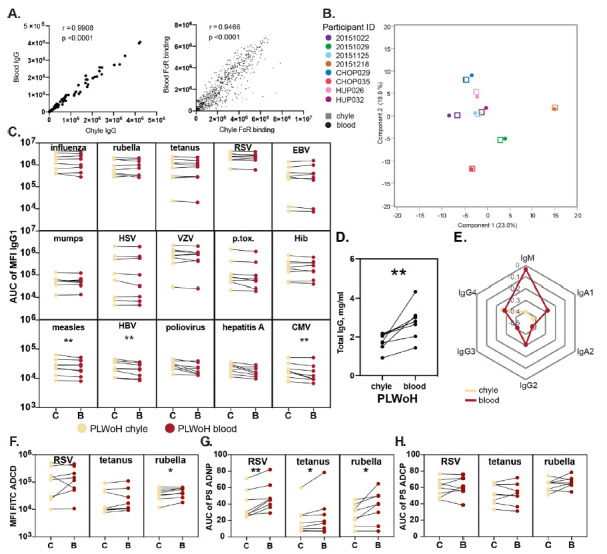
**Antibody levels and functions are similar between chyle and blood in people living without HIV (PLWoH).** (A) The Correlation plots show the Spearman correlation coefficient (r) between antigen-specific levels of IgG (left) and FcR binding (right) across matched blood and chyle pairs from n=8 PLWoH (B) Principal Components Analysis plot shows minor separation of antibody profiles for between chyle (squares) and blood (circles). Components include relative antibody isotype and subclass levels, FcR binding measurements, and effector function values (ADCP, ADNP, ADCD) for 15 antigens. (C) The paired line graphs show relative levels of antigen-specific IgG1 between the chyle (C) and blood (B). Data points represent the area under the curve of the median fluorescence intensity (MFI) from 3 sample dilutions (1:100, 1:500, 1:1000) for each individual and are an average of duplicate runs. (D) The paired line graph shows total immunoglobulin (IgG) titers, reported as mg/mL, in matched chyle and blood samples. Dots represent the mean of duplicate runs. (E) The radar plot shows median z-scored data (mean-centered and scaled to have unit variance) for relative levels of antibody isotypes and IgG subclasses across all antigens between chyle and blood. (F) The paired line graphs show antibody-dependent complement deposition (ADCD) between chyle and blood, depicted as the mean fluorescence intensity (MFI) of FITC, representing the deposition of C3 on antigen-conjugated beads coated with immune complexes for 3 representative antigens. Data points represent area under the curve of the MFI from 3 sample dilutions (1:10, 1:50, 1:250) for each patient and are an average of duplicate runs. (G, H) The paired line graphs show antibody-dependent neutrophil phagocytosis (ADNP) (G) and cellular phagocytosis (ADCP) (H) compared between chyle and blood for 3 representative antigens. Phagocytosis is measured by a phagocytosis score (PS), and data points represent the area under the curve for the PS over 3 sample dilutions (1:25, 1:100, 1:400) for each patient as an average of duplicate runs. Significance reported as *P<0.05, **P<0.01.

Striking correlations were observed in total IgG titers and FcR binding levels (Figure 1A) in the blood and the chyle across all tested individuals. Along these lines, multivariate profiling of anti-body levels and functions across all antigens demonstrated high concordance between blood and chyle across individuals rather than compartment-specific signatures (Figure 1B), irrespective of age and gender. Closer inspection of antigen-specific antibody levels across antigens demonstrated similar levels of IgG1 responses across most antigens (Figure 1C). However, slightly higher levels of measles-, hepatitis B virus-, and CMV-specific IgG1 levels were found in the chyle compared with the blood. Conversely, total IgG levels were significantly higher in the blood (Figure 1D), pointing to selective transfer of particular antigen-specific IgG antibodies into tissues. Together, these data suggest an effective transfer of antibodies between these 2 compartments rather than 2 independent selective compartments in terms of antibody composition.

We next aimed to define whether quantitative differences may exist in other antibody isotypes and subclasses across the blood and chyle. Isotype/subclass Luminex measurements demonstrated strong concordance in IgG2, IgG3, IgG4, and IgA2 levels across the compartments (Figure 1E). Conversely, chyle exhibited markedly lower IgM and IgA1 levels compared with the blood, highlighting isotype specific production and retention of IgM and IgA1 at the gastrointestinal mucosa, pointing to differential transfer of isotype to the chyle.

Given the differences in isotype-specific transfer, we next examined whether antibody function was transferred differentially to tissues. Limited differences were observed in RSV- and tetanus-specific antibody-mediated complement deposition across the chyle and blood (Figure 1F). Conversely, significantly higher rubella-specific complement deposition (ADCD) was observed in the blood, potentially linked to the differential utilization of IgM, excluded from tissues, to drive complement activation (Figure 1D). Antibody-dependent neutrophil phagocytosis (ADNP) was higher across all tested antigens in the blood (Figure 1G), potentially related to elevated levels of IgA1 in the blood known to trigger neutrophil activity in an Fcα-receptor-dependent manner [36]. In contrast, no differences were observed in antibody-dependent monocyte phagocytosis (ADCP) across the compartments, (Figure 1H) largely driven by IgG levels that appear to be equally distributed across the chyle and blood. These data highlight isotype-specific functional differences across the compartments, consistent with the dominant transfer of IgG, likely via FcRn, into tissues under normal homeostatic states.

### Reduced Transfer of Antigen-Specific IgG to the Chyle in people living with HIV

HIV infection, even on anti-retroviral therapy, is marked by persistent inflammation and altered immunity [37]. While mounting evidence suggests the cellular immune response is irreparably changed in PLWH, less is known about changes in antibody levels and distribution after HIV acquisition. With the growing interest in the use of monoclonal therapeutics for the treatment and potential functional eradication of HIV [38–47], understanding the impact of HIV infection on antibody distribution may have important implications for these new therapeutic approaches.

**Figure 2.**
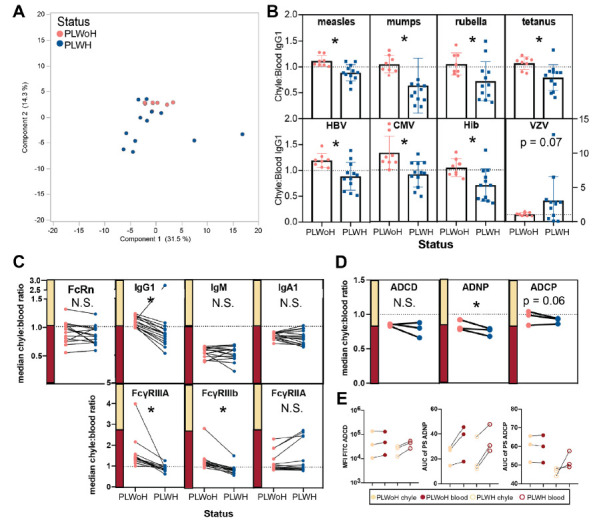
**Balance of antibody profiles between chyle and blood is disrupted in HIV infection.** (A) Principal Components Analysis plot highlights the distinction between PLWH (n=12) or PLWoH (n=8) patients for ratios of antibody isotypes, subclasses, FcR binding, and functions between chyle and blood. Negative PCA scores are indicative of a lower transfer ratio. (B) The bar graphs show chyle to blood ratios of relative IgG1 levels compared between PLWH or PLWoH. Dots represent the mean ± SD of duplicate runs for ratios of the area under the curve for MFI values at 3 sample dilutions for each individual. HBV = hepatitis B virus, CMV = cytomegalovirus, Hib = Haemophilus influenza b, and VZV = Varicella zoster virus. (C) Paired line graphs show the change of median chyle:blood ratios for 15 antigens between PLWH or PLWoH for levels of (top) FcRn, IgG1, IgM, IgA1, and (bottom) FcyRIIIA, FcyRIIIb, and FcyRIIA. (D) Antibody-dependent cellular phagocytosis (left), antibody-dependent neutrophil phagocytosis (middle), and antibody-dependent complement deposition (right). (E) Paired line graphs show antibody-dependent complement deposition (left), antibody-dependent neutrophil phagocytosis (middle), and antibody-dependent cellular phagocytosis (right) between chyle and blood for PLWoH (closed circles) and PLWH (open circles). Points represent the median area under the curve of either MFI or PS for RSV, rubella, and tetanus across all individuals. Significance for univariate analyses reported as, * *P*<0.05, N.S. = not significant.

We characterized antibody profiles across the blood and chyle in a second cohort of 12 PLWH who ranged in age from 23 to 64 years. Multivariate profiling of chyle: blood transfer ratios highlighted tissue-specific differences between those living with HIV (blue) and without HIV (pink). Profiling of transfer ratios revealed that PLWH clustered lower and away from PLWoH (Figure 2A), highlighting differences in antibody transfer across the blood:chyle in the context of HIV infection. To more closely analyze antibody distribution between chyle and blood across different antigen-specificities, we compared chyle:blood IgG1 ratios between all participants. Notably, across all antigens (except for varicella zoster), ratios were higher in PLWoH individuals without HIV compared to PLWH (Figures 2B).

Specifically, almost all individuals without HIV had ratios over 1, indicating higher antigen-specific IgG1 levels in chyle compared with blood. In contrast, most individuals with HIV had ratios below 1, where antigen specific IgG1 levels were higher in the blood (Figures 2B). Given that HIV infection is associated with hypergammaglobulinemia in the blood, these data point to a regulation of antibody transfer into tissues.

Further analysis of transfer ratios across isotypes highlighted more similar patterns in IgG subclass profiles across the group, but striking differences in FcR binding profiles between PLWH or PLWoH (Figure 2C). However, at a univariate level, significant differences were observed in IgG1 transfer in PLWH. Conversely, IgM and IgA1 transfer ratios were lower and stable irrespective of HIV status, with higher levels consistently observed in the blood (lower ratio, Figure 2C). Moreover, fewer differences were noted in subclass distribution across the groups (Figure 2C), unconfounded by hypergammaglobulinemia in HIV infection (Supplementary Figure 1).

Given the significantly reduced IgG transfer in HIV infection, we hypothesized that altered anti-body transfer may be related to changes in antibody binding to the neonatal FcR, FcRn. However, no differences were noted in overall FcRn transfer in PLWH (Figure 2C, left). This suggested that either hypergammaglobulinemia alone accounted for the striking variation in antibody transfer across tissues, or that alterations in IgG transfer may be related to other biological mechanisms of antibody transport.

Endothelial cells, like immune cells, can upregulate FcRs, enabling them to capture IgG with different affinities and mediate transfer across compartments [48]. Thus, we probed the levels of FcγR binding across the blood and chyle. Surprisingly, for both FcγRIIIA and FcγRIIIB, but not FcγRIIA, lower levels of FcγR binding antibody transfer were observed in PLWH (Figure 2C, bottom), suggesting that FcγRIIIs may contribute to altered antibody transfer in the context of HIV. Similarly, functional antibody levels were lower in the chyle of PLWH compared to the levels in PLWoH (Figure 2D), with slight differences in ADCD and ADCP and larger differences in ADNP. Additional functions, including NK cell activation, dominantly regulated by FcγRIII could not be investigated due to sample limitations. Moreover, functional ADCD antibody levels were generally lower in PLWH (Figure 2E), suggesting that while antibody-specificities were conserved, HIV infection is associated with reduced functional IgG transfer, which is likely governed by changes in Fc-mediated transfer into the chyle.

### FcγRIII-Driven Changes in Antibody Transfer in the context of HIV

**Figure 3.**
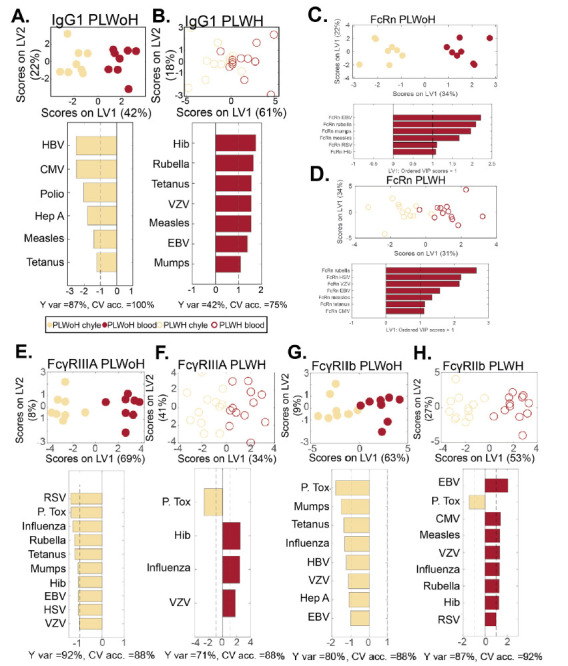
**Multivariate analysis of shift in IgG1 transfer to chyle in HIV infection.** Computational analysis was performed to identify the determinants of chyle and blood antibody profile differences. A multilevel PLSDA (MLPLSDA) modeling approach was used to define the specific features that most effectively provided resolution between chyle and blood for PLWoH (closed circles) and PLWH (open circles) based on (A, B) IgG1 (C, D) FcRn IgG1 (E, F), FcγRIIIA, and (G, H), and FcγRIIIB. Top plots of all panels depict the scores plots, where dots represent individual samples. The orthogonalized approach ensured that latent variable 1 (LV1) captured the separation between chyle and blood antibody profiles, whereas LV2 captured the antibody profile variances that do not contribute to this resolution. The bar graphs depict Variable Importance in the Projection (VIP) scores on LV1. Depicted are the prime factors defined as the factors that have VIP scores >1, indicating greater than average contribution to the separation of chyle and blood profiles. Factors pointing towards the left depicted in khaki are enriched in chyle, while those pointing towards the right in dark red are enriched in blood. Measures of model performance including cross-validated accuracy (CV acc.) for these models are reported in percentages below the bar plots. Furthermore, these models outperformed random label-shuffled models (Wilcoxon *P* = 0.02, 0.07, 0.04, 0.07, 0.03, 0.06, 0.03, 0.005 for the eight models of A-H, respectively). Y var depicts the percentage of variance in the separation variable Y accounted for by LV1.

To gain a deeper sense of the antibody profile differences across the chyle and blood in the context of HIV, a Multi-Level Partial Least Squares Discriminant Analysis (MLPLSDA) [31] model was developed, which took advantage of the paired structure of the data (paired chyle and blood samples). This analysis subtracted the effect of heterogeneity between chyle:blood samples (inter-pair variability) and focused on the effects within chyle:blood samples (Figure 3). We initially focused on FcRn-mediated transfer profiles, to determine whether changes in pathogen-specific FcRn levels differed across PLWoH. Limited differences were observed in FcRn transfer models across the two groups, both of which demonstrated significant differences between chyle and blood, suggesting that differential binding to this receptor could not account for differential transfer into tissues observed in PLWH or PLWoH (Figure 3A-D).

Specifically, similar antigen-specific antibody FcRn binding levels separated the chyle and blood profiles (EBV, rubella, mumps, etc), pointing to shared FcRn-mediated antibody transfer mechanisms across the 2 groups of individuals. Despite this lack of difference, antigen-specific antibody transfer was perturbed in the HIV-infected subjects (Figure 2), and shifted toward higher IgG1 in blood in blood from PLWH, for a slightly different set of antigens (Figures 2B).

Given the potential role of FcγRIII in altered IgG transfer across PLWH or PLWoH (Figure 2C and E), we next built models for the transfer of IgG and individual FcR within each patient group (Figure 3A-B). Striking differences were noted in IgG transfer across the two groups (Figure 3C and D), marked by antigen-specific IgG1 in the chyle in PLWoH, but a greater enrichment of antibodies in the blood of PLWH, marked by many shared immunodominant antigen-specificities. Similarly, analysis of FcγRIIIA binding profiles pointed to a robust enrichment of FcγRIIIA binding antibodies in the chyle of PLWoH (Figure 3E and F), but a predominant enrichment of antigen-specific FcγRIIIA binding antibodies in the blood of PLWH. Likewise, FcγRIIIB binding antibody profiles were enriched in the chyle of PLWoH, but enriched in the blood of PLWH (Figure 3G and H). Given the similarly divergent profiles of IgG and FcγRIII binding antibodies in the chyle and blood across the groups, these data highlight that changes in FcγRIII binding may contribute to altered antibody distribution across the compartments in HIV infection.

### HIV-Specific Responses are Lower in Chyle But Not Differentially Transferred Across Compartments.

**Figure 4.**
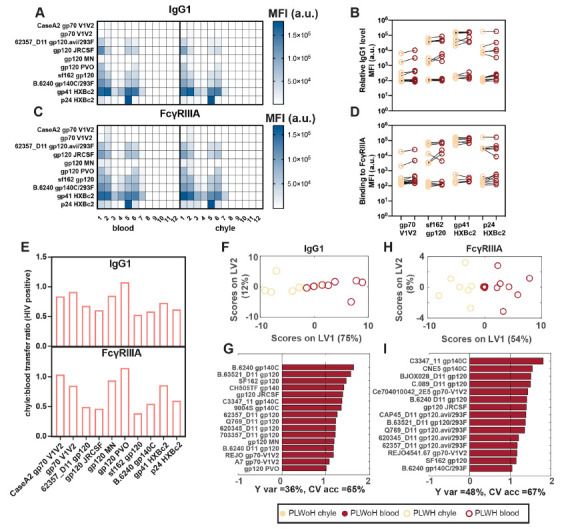
**Levels of HIV-specific IgG are not different between chyle and blood.** (A, C) The heat maps show relative levels of IgG1 (A) and FcγRIIIA (C) as measured by Luminex across different HIV epitopes. Shown on the right is a heatmap of mean fluorescence intensities (MFI) of the antigen. (B, D) The paired line graphs show the direct comparison between relative levels of IgG1 (B) and FcγRIIIA (D) between chyle and blood. (E) The bar graphs show the transfer ratios of IgG1 (top) and FcγRIIIA (bottom) between chyle and blood for HIV antigens. Each bar represents the median IgG1 and FcγRIIIA-binding across 12 PLWH. (F-I) The MLPLSDAs show the distinct separation between chyle and blood based on IgG1 (E, G) and FcγRIIIA (H, I) specific for HIV antigens (gp140, gp120, gp70, gp41, p24) in PLWH. (F, H) depict the scores plots, where dots are individual chyle and blood samples shown in khaki (chyle) and dark red (blood). (G, I) The Variable Importance in Projection (VIP) scores demonstrating the top antigen-specific IgG1 (G) and FcγRIIIA (I) associated with the differences between the antibody responses in chyle and blood, *i.e.* features with VIP scores > 1, indicating greater than average contribution. Factors in red pointing to the right are enriched in the blood. Cross-validated accuracy (CV acc.) for these models are reported in percentages below the bar plots. Furthermore, these models outperformed random label-shuffled models (Wilcoxon p = 0.17 and 0.08 for IgG1 and FcγRIIIA, respectively).

Given the differences in vaccine/endemic pathogen-specific antibody levels across the blood and chyle in PLWH, we next examined whether HIV-specific antibody transfer was also perturbed. IgG1 levels (Figure 4A and B) and FcγRIIIA binding (Figure 4C and D) across several HIV antigens were highly coordinated in chyle and blood. However, IgG transfer ratios across HIV envelope variable loops, the whole envelopes of different viral clades, gp41, and matrix p24 protein were consistently at or below a transfer ratio of 1 (Figure 4E), suggesting that higher concentrations of antibodies were consistently present in the blood. Moreover, these data indicate that despite the systemic hypergammaglobulinemia that persists throughout HIV infection, antibodies do not flow freely into the tissue, and that tight regulation of antibody transfer occurs between blood and tissue. Additionally, FcγRIIIa specific transfer was highly concordant with IgG1 transfer profiles, but were compromised for a specific subset of antigen-specific antibody subpopulations, again pointing to a potential role for FcγRIII in regulating antibody transfer differences. To examine this more closely, MPLSDA was used to examine compartment-specific differences.

Differences in antibody profiles between chyle and blood were driven by higher relative levels of IgG1 and FcγRIII-binding in the blood for all HIV-specific antibodies (Figures 4F-I). These data highlight lower antibody levels in chyle but a highly concordant transfer of HIV-specific antibodies across the compartments. Thus overall, these data point to significant and conserved isotype and FcγRIII-mediated sieving of antibodies across blood and tissues in health and disease, that may be leveraged in next-generation therapeutic design.

## DISCUSSION

The study of antibody transfer between blood and tissues has been hampered by the logistics of sampling tissue-derived antibodies broadly in humans. Stochastic sampling via biopsies may lead to biased results and the capture of antibodies from digested tissues is uncertain to reveal the overall landscape of antibodies found in tissues. Therefore, few human studies have profiled the composition of antibodies in tissues, whether they are distinct from circulating antibodies, whether they can elicit effector functions, and whether they change with chronic disease. However, understanding humoral immune access in tissues has important implications, as many infectious diseases [22] and cancers [49] reside in tissues. Moreover, defining how antibodies traffic between blood and tissues is of utmost importance for monoclonal immunotherapies, whose targets may reside partially or entirely within tissues[50].

Here, we applied a unique sample set of matched human thoracic duct chyle and peripheral blood samples from PLWH or PLWoH. We investigated functional and qualitative antibody profiles within these 2 compartments based on levels of different antibody isotypes, subclasses, FcR binding, and effector functions across a range of HIV and non-HIV antigens. IgG was selectively transferred to the chyle from blood in PLWoH, uniformly across all antibody specificities, linked to equivalent ADCP, but reduced levels of IgM-mediated ADCD and IgA-driven ADNP, highlighting the sieving of antibody functions into the tissue. Critically, this balance was perturbed in PLWH, with reduced overall IgG transfer ratios, linked to disrupted FcR binding, rather than changes in FcRn binding, pointing to a specific profile of sieving into inflamed tissues.

While IgG was transferred clearly from the blood to tissues, IgM and IgA demonstrated poor to no transfer. These data suggest that IgM and IgA observed in tissue biopsies are largely, or exclusively, produced locally by infiltrating B cells, and that these antibodies may not exit the tissues once produced locally. The exclusion of these isotypes may limit the highly inflammatory activities of these antibody isotypes, which exhibit high affinity for their respective FcRs or complement [51, 52]. Unlike mammary tissue, which expresses high levels of the polymeric immunoglobulin receptor (pIgR), the majority of endothelial cells do not express a receptor able to capture IgA or IgM to drive transfer [53]. Thus, therapeutic approaches leveraging IgA and IgM as therapeutics must take into consideration their limited penetration into tissues.

While no univariate difference in FcγRIIA-binding antibodies was observed across compartments, significant univariate and multivariate differences were noted in FcγRIIIA that mirrored differential IgG transfer. These data point to a critical role for FcγRIII receptors in selecting antibodies able to transit into tissues. While previous data argued that FcRn alone drove antibody transfer across the placenta, recent work suggests that FcRn may collaborate with FcγRIIIA to selectively transfer antibodies of the greatest functional relevance to the maturing infant [12]. FcγRIIIA expression and colocalization with FcRn were observed on syncytial trophoblasts [12] that even increased with active infection [54].

A study showed endothelial cells from human breast and aortic tissue increased expression of low-affinity FcγRII receptors in response to inflammatory mediators [55]. However, whether FcγRIIIA is also expressed on endothelial cells, permitting antibody selection, or whether additional non-canonical FcRs may exist on these unique barriers to the tissues remains unknown. However, the sieving points to a common signature of antibody selection across endothelial cells. Further characterizations of this selective process, and how it may be modulated by HIV or other chronic diseases, can provide critical insights for improving tissue penetration of monoclonal therapeutics.

Alterations in antibody transfer to tissues in HIV infection may be related to the significant hypergammaglobulinemia observed in HIV infection that does not resolve with treatment [56]. Reduced transfer ratios observed in HIV infection presented here may simply reflect the presence of a threshold of antibody that is permitted to transfer into tissues that may not change even if systemic antibody levels increase. Conversely, HIV-associated hypergammaglobulinemia is also associated with the production of persistently altered antibody-glycosylation [57] that may interact differentially with FcRs, and potentially contribute to restricted antibody access into tissues. Also, despite reduced hypergammaglobulinemia after prolonged ART treatment, persistent changes in antibody glycosylations have been reported [57]. Hence, antibodies do not flow equally across compartments, but exhibit quantitative and qualitative selection across the compartments. Thus, these data further highlight the unique sieve that may exist at the level of antibody-mediated tissue penetration, where tissue transfer may not exceed a particular level, even in the setting of chronic infection and inflammation.

Given emerging interest in monoclonal therapy for HIV eradication, it is critically important to understand whether administered antibodies reach target sites where the virus resides. Data from the monoclonal therapeutics field has documented the incomplete depleting effects [58, 59]. These gaps in therapeutic benefit may be attributable to our incomplete understanding of how antibodies access tissues. Most studies focus on the role of FcRn-mediated antibody transfer through tissues [60–63]. Our findings point to the potential role of antibody-binding receptors, including FcγRIII receptors, in the capture and transfer of antibodies to tissues. Moreover, enhanced depletion of B cells has been observed using afucosylated variants of B cell depleting antibodies [64]. Fc-afucosylation is known to enhance monoclonal binding to FcγRIIIA [65]. However, whether afucosylation solely contributed to enhanced B cell clearance via enhanced activation of cytotoxic cells or also via enhanced tissue penetration remains unclear. However, given the rapid growth of monoclonal therapeutics for the treatment of a broad array of diseases, understanding the rules of antibody-mediated tissue penetration may provide novel opportunities to improve therapeutic outcomes and to fight diseases including HIV and beyond.

## LIMITATIONS

There are several limitations to the present study that must be acknowledged. While this study focused on the blood:chyle interface, and indirect measures of transfer, characterizing antibody transfer across mucosal surfaces would be highly informative, particularly for IgA subclasses. Moreover, despite this being a first-in-class study, we were limited by enrollment in the cohort analyzed. Thus, while the study size is small, it points to unique biology that may help inform future mAb therapies and the biology of antibody transfer from tissues. The cohort size limited our ability to analyze how HIV viral loads may impact trafficking; however, models accounting for blood:chyle antibody transfer using HIV viral copies/mL may reveal further insight into how the presence and abundance of the pathogen impact this phenomenon. Furthermore, heterogeneity was observed even in our relatively small sample size. Future work aimed at identifying the generalizability of the present study, particularly with regards to antibodies directed to specific targets, would benefit from a larger sample size that could provide important validating results. It is unlikely that hypergammaglobulinemia alone can explain the compartmentalization we observed, given that long-term ART status resolves hypergammaglobulinemia [66]. However, we cannot discount that hypergammaglobulinemia could be a significant contributor to altered antibody distribution in blood and tissues in untreated PLWH.

This study focused on antibody profiling in chyle, rather than individual tissues. Thus, whether all tissues are accessed by similar or distinct antibodies remains to be determined. Moreover, a select panel of representative antigens of common vaccines and high community-prevalent pathogens was used to assay for transfer across the blood:chyle barrier; further work into how lower-exposure rate antigens are affected by the phenomenon we observed is warranted. Lastly, discrete antigen-specific glycosylation profiles of antibodies could not be measured in this study because of sample volume size limitations. Antibodies possess distinct glycans depending on their antigen target, recency of antigen encounter [57, 67], geographic influences [68], and these glycans shape FcR interactions and could also influence transfer into tissues [69]. Also, even while on ART, humoral profiles can skew towards inflammatory signaling through glycosylation patterns of long duration [57], which may in turn impact antibody transport.

## SUPPLEMENTARY DATA

Supplementary materials are available at the Pathogens and Immunity website. Supplementary data may be provided by the authors to benefit the reader. Supplementary data are not copyedited and are the sole responsibility of the authors. Questions or comments related to supplementary materials should be addressed to the corresponding author.
