# Supplementary material for: Distinct Antibody Fc-profiles in Lymph During Homeostasis and Chronic HIV Infection
Source: Pathog Immun. 2026 Feb 23;11(1):111–34. doi: 10.20411/pai.v11i1.887 (PMC12952224; doi:10.20411/pai.v11i1.887)
Supplement: Supplementary Table and Figure [file pai-11-111-s01.pdf]

PATHOGENS AND IMMUNITY
IN ASSOCIATION WITH CASE WESTERN RESERVE UNIVERSITY AND THE RICHARD J. FASENMYER FOUNDATION

Supplementary Table 1. Antigen Reagents

Pathogen	Antigen	Vendor, Catalog #
Measles, Edmonston	Viral lysate	BioRad, PIP013
Mumps, Enders	Viral lysate	BioRad, PIP014
Rubella, HPV-77	Viral lysate	BioRad, PIP044
Varicella zoster virus (VZV)	gE(Orf68)	Made in house
Epstein barre virus (EBV)	gp350/220	Immune Technology, IT-005-035p
Herpes simplex virus (HSV)	gD (1), gC (2)	Immune Technology, IT-005-055p, IT-005-011p
Influenza A	HA H1N1 and H3N2	Immune Technology, IT-003-0042ΔTMp, IT-003-001p, IT-003-0011ΔTMp
Respiratory Syncytial virus (RSV)	Pre and post A and B	Barney Graham, NIH
Cytomegalovirus (CMV)	gH pentamer complex	CMV-PENT-100
Poliovirus	Polio vaccine salk inactive	MGH Pharmacy, 976210
Tetanus	Toxoid	UMass Medical Center
Pertussis, Bordetella	Toxin	List Biological Laboratories, #180
Hepatitis A	Vaccine, HAVRIX	MGH Pharmacy, 5SR75
Haemophilus influenzae B	Vaccine, Polysaccharide conj-tet	MGH Pharmacy, 2000696
Hepatitis B virus (HBV)	HBsAg adw	Genway, GWB-B11E0A

McNamara RP, Butler AL, Dolatshahi S, Taylor S, Dori Y, Frank I, Itkin MG, Betts MR, Alter G. Distinct Antibody Fc-profiles in Lymph During Homeostasis and Chronic HIV Infection. *Pathogens and Immunity*. 2025;10(2):111-134. doi: 10.20411/pai.v11i1.887

Supplementary Figure 1

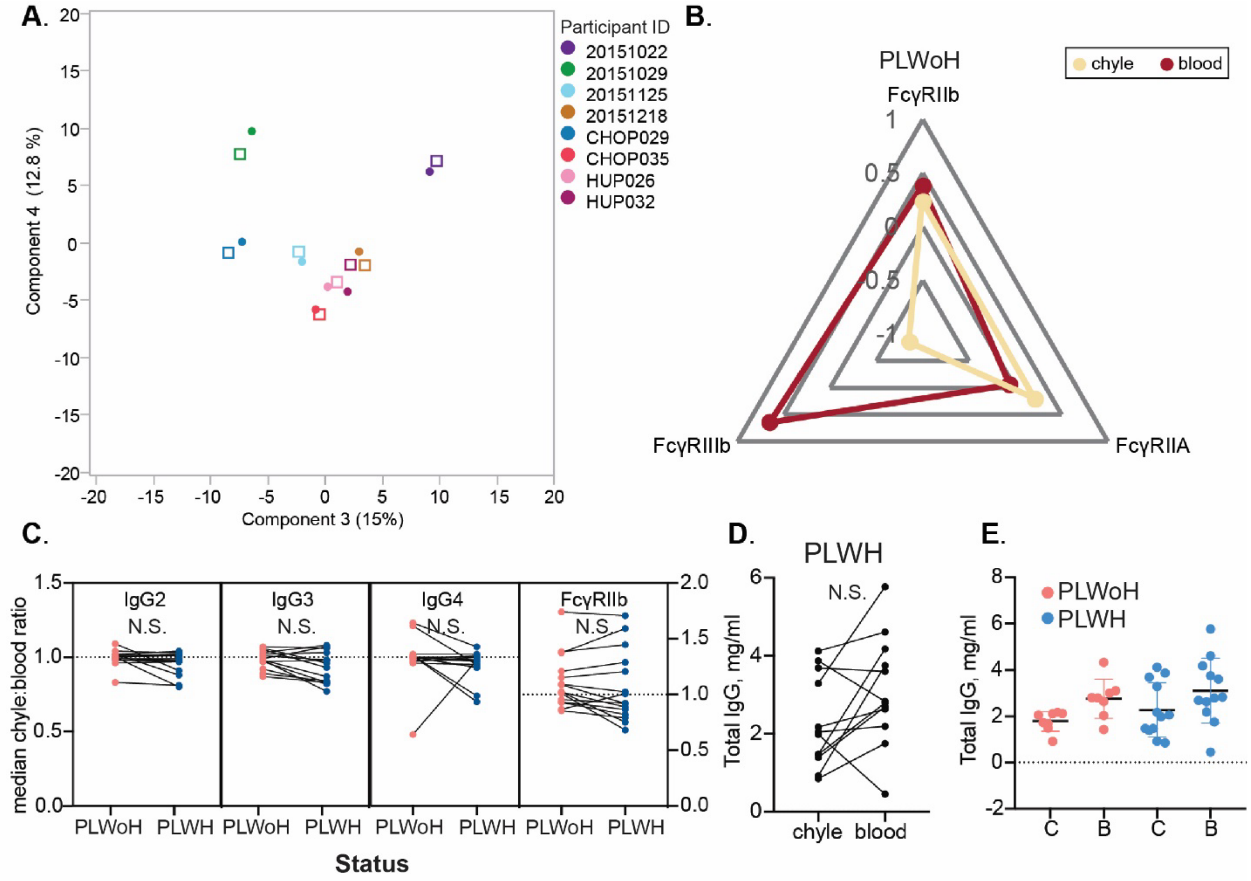

Supplementary Figure 1. A) Principal Components Analysis plot with components 3 and 4 shows no separation of antibody profiles for 8 persons living without HIV (PLWoH) between chyle (squares) and blood (circles) by PC3 and PC4. B) The radar plot shows median z-scored data for FcγR binding for RSV, rubella, and tetanus between chyle and blood from PLWoH participants. C) The paired line graphs show the change of median chyle:blood ratios for each antigen between PLWoH and persons living with HIV (PLWH) participants for levels of IgG2, 3, 4, and FcγRIIb. Each colored dot represents the median ratio for a specific antigen across all individuals within a patient group. D) Paired line graph shows total immunoglobulin (IgG) titers, reported as mg/ml, in matched chyle and blood samples from PLWH participants. Dots represent the mean of duplicate runs for 12 participants. E) The dot plot shows there are no significant differences in total IgG titer in chyle and blood between PLWoH and PLWH.

McNamara RP, Butler AL, Dolatshahi S, Taylor S, Dori Y, Frank I, Itkin MG, Betts MR, Alter G. Distinct Antibody Fc-profiles in Lymph During Homeostasis and Chronic HIV Infection. *Pathogens and Immunity*. 2025;10(2):111-134. doi: 10.20411/pai.v11i1.887
